# Awareness of the Risk of Chronic Use of Steroid Causing Cataract in Al Ahsa City, Saudi Arabia

**DOI:** 10.7759/cureus.52861

**Published:** 2024-01-24

**Authors:** Abdulaziz I AlSomali, Hajar M AlHajri, Rawabi Aljumaiah, Muntaha N Alnasser, Zainab Alabdullah

**Affiliations:** 1 Ophthalmology, King Faisal University, Al Hasa, SAU; 2 Medicine, King Faisal University, Al Hasa, SAU

**Keywords:** al ahsa, steroid, vision impairment, opacity, cataract

## Abstract

Introduction

The lens, essential for vision, can be impaired by cataracts, leading to partial or complete reversible vision loss. Common risk factors include aging, diabetes, and steroid use, with significant financial implications. Limited awareness in Saudi Arabia necessitates further research to reduce cataract prevalence and increase knowledge about steroid-induced cataracts.

Methodology

This was a cross-sectional study in Al Ahsa City, Saudi Arabia that aims to assess awareness of cataracts induced by long-term steroid use. Data was collected via an online survey and analyzed using Statistical Package for Social Sciences (SPSS) version 29 (IBM Corp., Armonk, NY, USA).

Results

Our study results show that 69.8% (n=291) of participants were female, and 30.2% (n=126) were male, with the majority (62.6%, n=261) having a university education. Notably, 91.1% (n=380) reported no steroid use, while 8.9% (n=37) reported long-term use, and 10.1% (n=42) used steroids topically. There are moderate awareness levels regarding cataract and steroid associations, with 68.1% (n=284) recognizing topical steroids as the common culprits. Logistic regression highlighted the positive correlation between knowledge of cataract risks due to steroid use and actual steroid use, corroborated by a notable 73.0% (n=27) steroid usage among high-awareness individuals.

Conclusion

Our study underscores moderate awareness regarding steroid-related cataract risks in Al Ahsa City. Educational status significantly influenced understanding, highlighting the importance of targeted health education initiatives.

## Introduction

The lens is an ellipsoid structure, a crystalline substance located in the eye. The lens's primary optical purpose is to transmit light and focus it on the retina. It is composed of certain architecture and cellular contents which are crucial for its transparency [1]. A cataract is an opacity of the lens that worsens the sight because of a decrease in the amount of incoming light [2].

A cataract can lead to visual deterioration, resulting in reduced vision capacity and partial or complete reversible vision loss [3]. In 2020, cataract was the leading cause of total blindness in adults aged 50 years and older with 45·5% of all cases [3]. A study conducted in Saudi Arabia between 1986 and 2015 shows that cataract was the leading cause of blindness and the second leading cause of moderate to severe vision impairment [3].

The most commonly associated risk factors for cataract include aging, diabetic retinopathy, glaucoma, and some medications such as glucocorticoids [4,5]. Glucocorticoids are utilized in a variety of disorders, as long- and short-term, treatment such as topical ocular steroid usage, allergic rhinitis and inflammatory bowel disease as a result, the chance of cataract may increase and put a significant burden in countries [6,7]. Using glucocorticoids either as inhaler and/or orally will increase the risk and prevalence of posterior subcapsular cataract and other ocular complications such as glaucoma [8-10]. Cataract treatment and care are quite expensive and constitute a financial burden, so it is crucial to prevent cataract risks in order to decrease the financial burden that cataract places on people and health authorities [11,12]. According to several studies, the Saudi population has poor knowledge regarding cataract and inadequate knowledge regarding steroid-induced cataract thus it is crucial to do further research in Saudi Arabia to elevate the level of awareness and reduce the prevalence of cataracts [13,14]. Our study aims to assess the awareness level of cataract and steroid-induced-cataract in Al Hasa in the east region of Saudi Arabia.

## Materials and methods

Study design and setting

This was a cross-sectional study that targeted the general population in Al Hasa City, Saudi Arabia to analyze the level of awareness of the cataract caused by long-term steroid use. The study was conducted between April 2023 and September 2023.

Study subjects, inclusion, and exclusion criteria

Subjects for the study included all people who live in Al Ahsa City, Saudi Arabia who consented to participate between April and September 2023 and who matched the inclusion and exclusion criteria. Adults in Al Ahsa, Saudi Arabia who were at least 18 years old, reside there, and have consented to participate in the study all met the requirements for inclusion. The exclusion criteria included being under the age of 18, living outside of Al Ahsa, and refusing to take part in the study.

Sampling and sample size

The Saudi public was asked to participate via an online link, and a convenient random sample was employed as the sampling approach. Social media outlets were used to distribute the questionnaire. The sample size was established using the equation n = z2pq\d 2. with an anticipated proportion of 50%, a level of 95% confidence, and a precision of 5%. A 385-person sample size was chosen as the minimum. More participants and candidates were included to ensure the sufficiency and accuracy of the outcomes.

Data collection and study tool

An online survey was taken from previously conducted research for data collection using Google Forms. The population in Al Ahsa was informed about the online poll and invited to participate. Participants must have consented to participate in the study before starting the online questionnaire that was used to gather data. The survey was divided into three key components, the first of which was focus on sociodemographic data such as age, sex, the region in Saudi Arabia, nationality, career-study major, and education level. The second part of the questionnaire inquired about the understanding of the prevalence and risk factors for steroid-induced cataracts. The third portion included a question about the effects and management of steroid-induced cataract.

Data management and statistical analysis

Statistical Package for Social Sciences (SPSS) version 29 (IBM Corp., Armonk, NY, USA) was used to analyze the data. The frequency and percentages used to display categorical variables. The phrases minimum, maximum, mean, and standard deviation will be used to portray numerical variables. To compare the variables, the chi-square test was used. The threshold for significance was fixed at 0.05. 

Confidentiality and ethical consideration

In managing data, the utmost level of confidentiality was applied. The confidentiality of all study participants was protected at all times. The ethics committee at the Deanship of Scientific Research at King Faisal University in Saudi Arabia was consulted for approval (approval number KFU-REC-2023-AUG-ETHICS1058).

## Results

Our study results involved 417 participants from Al Ahsa City, with a gender distribution of 69.8% female and 30.2% male. The age groups were as follows: 18-25 years (56.8%), 26-35 years (18.2%), 36-45 years (9.4%), and >45 years (15.6%). Educational status included primarily university-educated individuals (62.6%). Most participants were single (51.6%), and the majority (91.1%) did not use steroids, with 8.9% reporting long-term use and 10.1% using steroids topically as shown in Table 1.

**Table 1:** Sociodemographic and other parameters of participants.

		Frequency (n=417)	Percent
Gender	Female	291	69.8
	Male	126	30.2
Age	18-25 Years	237	56.8
	26-35 Years	76	18.2
	36-45 Years	39	9.4
	> 45 Years	65	15.6
Educational Status	Primary	6	1.4
	Medium	11	2.6
	High School	139	33.3
	University	261	62.6
Residence	Alahsa City	417	100.0
Marital Status	Single	215	51.6
	Married	193	46.3
	Widowed	9	2.2
Use Steroids (Cortisone) for Long Period	No	380	91.1
	Yes	37	8.9
Type of Steroid (Cortisone) Use	N/A	343	82.3
	Topical	42	10.1
	I/V	6	1.4
	Oral	19	4.6
	Inhaled	7	1.7

Table 2 shows an assessment of the knowledge regarding the relationship between the use of steroids and the development of cataracts. Notably, 42.9% correctly associated cataracts with an opacity of the inside lens of the eye, while 16.5% recognized the characteristic appearance of cataracts resembling waxed paper. Furthermore, 53.2% acknowledged that aging and family history are common risk factors for cataracts, and 68.8% understood that cataracts can lead to vision deterioration. Additionally, 65.0% recognized that untreated cataracts could result in complete blindness. Concerning the awareness of the association between chronic steroid usage and ocular effects, including cataracts, 36.5% acknowledged this connection. 68.1% recognized topical steroids as the most common type associated with cataracts. Regarding the type of steroids, 22.5% recognized that oral and intravenous steroids may increase the incidence of cataracts, albeit to a lesser extent compared to topical steroids. Furthermore, the awareness that skin diseases such as atopic dermatitis (AD) and allergic contact dermatitis, which often require long-term steroid therapy, can elevate the risk of cataracts was acknowledged by 30.2% of the respondents. Additionally, 36.2% recognized that chronic steroid use could lead to increased intraocular pressure. Moreover, there was moderate awareness (27.6%) that steroid-induced cataracts can be distinguished from other types of cataracts. Notably, a substantial proportion (61.4%) acknowledged that steroid-induced cataracts could be treated with surgery, indicating a relatively better understanding of treatment options. 

**Table 2:** Assessment of knowledge about use of steroids causing cataract.

		Don't Know	No	Yes
Cataract is an opacity of the inside lens of the eye	N	208	30	179
	%	49.9	7.2	42.9
Cataract is described as looking like waxed paper	N	293	55	69
	%	70.3	13.2	16.5
Aging and family history are the most common risk factors for cataracts	N	167	28	222
	%	40.0	6.7	53.2
Cataract is associated with deterioration of the vision	N	106	24	287
	%	25.4	5.8	68.8
Cataracts could result in complete blindness if left untreated	N	120	26	271
	%	28.8	6.2	65.0
Chronic usage of steroids is associated with ocular effects including glaucoma and cataract	N	244	21	152
	%	58.5	5.0	36.5
Topical steroid is the most common type associated with cataract	N	284	43	90
	%	68.1	10.3	21.6
Oral and intravenous steroids could increase the incidence of cataracts but with lower association than topical steroids	N	295	28	94
	%	70.7	6.7	22.5
Skin diseases such as atopic dermatitis (AD) and allergic contact dermatitis are chronic, and necessities long-term steroid therapy thus increasing the risk of cataracts	N	238	53	126
	%	57.1	12.7	30.2
Chronic steroids could result in intraocular pressure	N	240	26	151
	%	57.6	6.2	36.2
Steroid-induced cataracts can be distinguished from other types of cataracts	N	258	44	115
	%	61.9	10.6	27.6
Steroid-induced cataracts could be treated with surgery	N	142	19	256
	%	34.1	4.6	61.4

Table 3 shows the result of logistic regression analysis examining predictors of chronic steroid users. Significant predictors include high school education with a negative association and adjusted odds ratio (aOR) of 0.051 (95% CI: 0.00-0.33), university education with a negative association and aOR of 0.080 (95% CI: 0.01-0.48), and knowledge about cataract due to steroid use with a positive association and an aOR of 1.062 (95% CI: 1.00-1.11). Other predictors like gender, age group, marital status, and education (medium) did not show significant associations.

**Table 3:** Adjusted predictors of chronic steroid users (logistic regression analysis). aOR: adjusted odds ratio

	B	Sig.	aOR	95% CI	
				Lower	Upper
Gender (Male)	.084	.829	1.088	.50	2.33
Age (18-25 Years)	Ref	.404	Ref		
Age (26-35 Years)	.285	.591	1.330	.47	3.75
Age (36-45 Years)	.263	.733	1.301	.28	5.89
Age (> 45 Years)	.954	.098	2.595	.83	8.02
Education (Primary)	Ref	.023	Ref		
Education (Medium)	-21.762	.999	.000	.00	
Education (High)	-2.981	.002	.051	.00	.33
Education (University)	-2.532	.006	.080	.01	.48
Marital Status (Single)	Ref	.499	Ref		
Marital Status (Married)	-.422	.390	.656	.25	1.71
Marital Status (Widowed)	.469	.622	1.599	.24	10.31
Knowledge about Cataract due to Steroid Use	.060	.023	1.062	1.00	1.11
Constant	-.520	.569	.594		

Table 4 shows the association between awareness of chronic steroid use and sociodemographic parameters. Significant associations are observed with the use of steroids (p = 0.003) and type of steroid (p < 0.001). High-level awareness is linked with a higher proportion of individuals using steroids (73.0%) compared to those not using steroids (47.6%). Furthermore, it shows a notable association with the type of steroids, particularly in the inhaled category, where 100% of those with high-level awareness use this type.

**Table 4:** Association of awareness of chronic steroid use with sociodemographic parameters.

			Awareness about Chronic Steroids causing cataracts		Sig. Value
			Low-Level Awareness	High-Level Awareness	
Gender	Female	N	151	140	0.272
		%	51.9%	48.1%	
	Male	N	58	68	
		%	46.0%	54.0%	
Age	18-25 Years	N	117	120	0.962
		%	49.4%	50.6%	
	26-35 Years	N	40	36	
		%	52.6%	47.4%	
	36-45 Years	N	19	20	
		%	48.7%	51.3%	
	> 45 Years	N	33	32	
		%	50.8%	49.2%	
Education	Primary	N	5	1	0.467
		%	83.3%	16.7%	
	Medium	N	6	5	
		%	54.5%	45.5%	
	High School	N	68	71	
		%	48.9%	51.1%	
	University	N	130	131	
		%	49.8%	50.2%	
Marital Status	Single	N	110	105	0.829
		%	51.2%	48.8%	
	Married	N	94	99	
		%	48.7%	51.3%	
	Widowed	N	5	4	
		%	55.6%	44.4%	
Use of Steroids	No	N	199	181	0.003
		%	52.4%	47.6%	
	Yes	N	10	27	
		%	27.0%	73.0%	
Type of Steroid	N/A	N	191	152	<0.001
		%	55.7%	44.3%	
	Topical	N	15	27	
		%	35.7%	64.3%	
	I/V	N	1	5	
		%	16.7%	83.3%	
	Oral	N	2	17	
		%	10.5%	89.5%	
	Inhaled	N	0	7	
		%	0.0%	100.0%	

## Discussion

The lens, a crucial structure in the eye, facilitates vision by focusing light on the retina. Cataracts, characterized by lens opacities, often lead to vision impairment, with aging and glucocorticoid use among the significant risk factors. Cataract poses a considerable financial burden on healthcare systems. Our study in Al Ahsa City, Saudi Arabia, focused on understanding the local population's awareness of the link between chronic steroid use and cataract development. Our discussion highlights key findings, including participant demographics, cataract awareness, the steroid-cataract connection, and factors influencing steroid use.

Regarding the sociodemographic features, our study contains predominantly female (69.8%) and relatively young population, as 56.8% belonged to the 18-25 years age group. Thus socio-demographic profile reflects similarities with previous research, demonstrating a trend of predominantly female participants and a relatively young population, in line with broader healthcare participation patterns and more prevalence of cataracts in females. Moreover, Lou et al. (2018) show that percentages of blindness due to cataracts among women and men were 35.5% and 30.1%, respectively [15]. The high proportion of university-educated individuals aligns with previous studies emphasizing the influence of education on health literacy. The prevalence of single individuals may indicate the impact of lifestyle factors on healthcare decisions. Notably, the significant proportion not using steroids aligns with global trends, underscoring the need for further research to understand the factors influencing steroid use patterns in different populations.

Regarding the participants' knowledge of cataracts and their association with steroid use, our findings revealed some encouraging levels of awareness. There were also areas where gaps in knowledge were evident. A substantial 42.9% correctly associated cataracts with an opacity of the inside lens of the eye. Similarly, Samuel et al. (2021) show that 88.4% of participants answered the correct simple definition of cataract [16]. While 68.8% of participants understood that cataracts can lead to vision deterioration consistent with the finding by Fateen et al. (2017), which stated that cataract is a principal cause of vision loss in the world [17]. Also, 65.0% recognized that untreated cataracts could result in complete blindness [18]. However, there were notable gaps in understanding. Only 16.5% recognized the characteristic appearance of cataracts resembling waxed paper. While, 36.5% acknowledged the association between chronic steroid usage and ocular effects, including cataracts. A systemic review by Black et al. (2016) stated a possible association between glucocorticoid use and the development of cataract [19].

There are varying awareness levels regarding the association between different types of steroids and cataract development. While 68.1% recognized the link with topical steroids [20], only 22.5% were aware of the impact of oral and intravenous steroids. Moreover, 30.2% acknowledged the elevated risk of cataracts due to long-term steroid therapy for certain skin conditions as indicated by Bair et al. (2011) showing cataract development in atopic dermatitis due to steroid usage [21].

Furthermore, a substantial proportion (61.4%) acknowledged that steroid-induced cataracts could be treated with surgery. Similarly, Sutyawan et al. (2019) highlight the treatment of choice is lens extraction with or without intraocular lens (IOL) for steroid-induced cataract [22]. This demonstrates a relatively better understanding of the available treatment options for steroid-induced cataracts.

Among the study's participants, high school and university education were negatively associated with chronic steroid use (aOR: 0.051 and 0.080, respectively), indicating lower usage in individuals with higher education. In contrast, knowledge about cataract due to steroid use was positively associated (aOR: 1.062), suggesting greater use in those aware of the steroid-cataract connection. No significant associations were found for gender, age groups, marital status, or medium-level education.

There is a notable association with inhaled steroids and heightened awareness might reflect the specific healthcare practices or educational initiatives targeting certain patient populations. Comparatively, previous literature has emphasized the importance of educational campaigns in enhancing patient awareness and shaping medication adherence patterns, highlighting the need for tailored educational interventions to improve patient understanding of medication-related risks and benefits.

Limitations

This was a cross-sectional design study and used an online disseminated questionnaire to collect the data which may have an impact on the accuracy of the results. However, this study provides a useful baseline for the awareness regarding steroid-related cataract risks in Al Ahsa city. Moreover, this study's sample size is considered to be high which more accurately reflects the bias-free status. 

## Conclusions

Our study underscores the varying levels of awareness regarding the risks associated with chronic steroid use and cataract development in Al Ahsa City, Saudi Arabia. The findings emphasize the crucial role of education and knowledge in influencing health-related behaviors. Tailored educational interventions are imperative to enhance awareness, particularly among populations at risk, ultimately facilitating informed decision-making and improving overall health outcomes.
